# Do daily fluctuations in inhibitory control predict alcohol consumption? An ecological momentary assessment study

**DOI:** 10.1007/s00213-018-4860-5

**Published:** 2018-03-01

**Authors:** Andrew Jones, Brian Tiplady, Katrijn Houben, Chantal Nederkoorn, Matt Field

**Affiliations:** 1 Department of Psychological Sciences, University of Liverpool, Liverpool, L69 7ZA UK; 2 UK Centre for Tobacco and Alcohol Studies, Liverpool, UK; 3 Anaesthesia, Intensive Care and Pain Medicine, University of Edinburgh, Edinburgh, UK; 4 Mobile Cognition Ltd., Edinburgh, UK; 5 Faculty of Psychology and Neuroscience, Maastricht University, Maastricht, The Netherlands

**Keywords:** Alcohol, Craving, Ecological momentary assessment, Inhibitory control, Stop signal task

## Abstract

**Rationale:**

Deficient inhibitory control is predictive of increased alcohol consumption in the laboratory; however, little is known about this relationship in naturalistic, real-world settings.

**Objectives:**

In the present study, we implemented ecological momentary assessment methods to investigate the relationship between inhibitory control and alcohol consumption in the real world.

**Methods:**

Heavy drinkers who were motivated to reduce their alcohol consumption (*N* = 100) were loaned a smartphone which administered a stop signal task twice per day at random intervals between 10 a.m. and 6 p.m. for 2 weeks. Each day, participants also recorded their planned and actual alcohol consumption and their subjective craving and mood. We hypothesised that day-to-day fluctuations in inhibitory control (stop signal reaction time) would predict alcohol consumption, over and above planned consumption and craving.

**Results:**

Multilevel modelling demonstrated that daily alcohol consumption was predicted by planned consumption (*β* = .816; 95% CI .762–.870) and craving (*β* = .022; 95% CI .013–.031), but inhibitory control did not predict any additional variance in alcohol consumption. However, secondary analyses demonstrated that the magnitude of deterioration in inhibitory control across the day was a significant predictor of increased alcohol consumption on that day (*β* = .007; 95% CI .004–.011), after controlling for planned consumption and craving.

**Conclusions:**

These findings demonstrate that short-term fluctuations in inhibitory control predict alcohol consumption, which suggests that transient fluctuations in inhibition may be a risk factor for heavy drinking episodes.

**Electronic supplementary material:**

The online version of this article (10.1007/s00213-018-4860-5) contains supplementary material, which is available to authorized users.

## Introduction

Inhibitory control—the ability to stop, change or delay inappropriate behaviour(s) (Logan et al. 1984)—is a fundamental component of impulsivity and executive functioning (Bickel et al. 2012) and an important component of the broader construct of self-control (Duckworth and Kern 2011; Fujita 2011). Inhibitory control can be operationalised in the laboratory using computerised tasks such as the stop signal task. This task requires participants to make rapid manual responses to arbitrary ‘go’ stimuli that appear on the screen. On a minority of trials, a visual or auditory ‘stop’ signal is presented a short time after presentation of the go stimulus, and this signals to participants that they should inhibit their response. On stop trials, participants’ behaviour can be characterised as a ‘race’ between the motor response and the inhibition of that response (Band et al. 2003). Deficits in performance on this task (and conceptually related tasks, such as the go/no-go and Flanker tasks) have been observed in alcohol-dependent patients (compared to healthy controls: Smith et al. 2014). Within non-dependent alcohol consumers, inhibitory control is worse in those who drink more heavily (Christiansen et al. 2012b; Houston et al. 2014; Smith et al. 2014), and it is associated with ad libitum alcohol consumption in the lab (Jones et al. 2013b). Longitudinal studies have demonstrated that inhibitory control predicts progression from heavy drinking to dependence (Rubio et al. 2008) and the likelihood of relapse following treatment (Rupp et al. 2016). Furthermore, the development of inhibitory control during childhood and adolescence is closely linked to the initiation and escalation of substance use, including alcohol consumption (Fernie et al. 2013; Nigg et al. 2006). Finally, the capacity for inhibitory control may be essential for resisting temptation (Fujita 2011; Hofmann et al. 2009): Deficits in inhibitory control may make substance users more likely to engage in substance use, even if they are attempting to abstain.

Despite the presence of between-group differences, laboratory research suggests that within alcohol consumers, inhibitory control is not a stable trait. Rather, it appears to fluctuate in response to internal and environmental events (De Wit 2009; Jones et al. 2013a) such as stress (Zack et al. 2011), depletion of self-control resources (Muraven et al. 2002) and exposure to alcohol-related cues or contexts (Czapla et al. 2015; Jones and Field 2015). These momentary fluctuations in inhibitory control during such ‘high-risk’ situations may increase the likelihood that people will drink alcohol (De Wit 2009; Jones et al. 2013a). Consistent with this view, laboratory studies demonstrate that experimental manipulations of disinhibited mindsets can influence subsequent ad libitum drinking behaviour (Jones et al. 2011a; Jones et al. 2011b). In these studies, participants who were required to prioritise successful inhibition over rapid responding as they completed a stop signal task subsequently consumed less alcohol than participants who were given standard instructions (i.e. to give equal priority to rapid responding and successful inhibition) before they completed the task. These studies suggest a causal relationship between state fluctuations in inhibitory control and alcohol consumption soon afterwards, when both are measured in the laboratory (see also Field and Jones 2017). However, little is known about the predictive relationship between fluctuations in inhibitory control and alcohol consumption in naturalistic settings, outside of the laboratory.

Ecological momentary assessment (EMA) methods are particularly well suited for investigation of the precursors and triggers of substance use in real-world settings (Shiffman 2009; Shiffman et al. 2008). EMA involves repeated sampling of participants’ environmental context, subjective states and behaviour using mobile devices (typically smartphones or personal digital assistants (PDAs)) as they go about their lives. EMA studies in the addiction field have been particularly useful for identifying the temporal relationships between exposure to substance-related cues, fluctuations in subjective craving and substance use (Fatseas et al. 2015; Serre et al. 2015). Furthermore, daily assessment of alcohol intake using EMA yields more reliable estimates of consumption than conventional retrospective diary measures (Carney et al. 1998; Monk et al. 2015; Searles et al. 2000). Importantly, EMA methods have recently been implemented to objectively assess cognitive precursors of substance use, such as attentional biases, in naturalistic settings (Marhe et al. 2013; Waters et al. 2012).

To date, EMA methods have not been applied to investigate associations between objectively measured inhibitory control and alcohol consumption, including ‘limit violations’ (drinking more alcohol than planned (Collins et al. 1994; Muraven et al. 2005a)). In order to investigate this issue, it is important to study individuals who are motivated to restrict their alcohol intake and are currently attempting to do so, on the basis of theoretical claims that the predictive power of inhibitory control for consumptive behaviour should be greatest amongst those who are attempting to control that behaviour, and therefore likely to attempt to engage inhibitory control in order to do so (Hofmann et al. 2012; Wiers et al. 2010). Indeed, laboratory studies that investigated the association between inhibitory control (and related constructs, such as subjective self-control) and alcohol intake deliberately motivated participants to restrict their drinking before assessing alcohol intake (Christiansen et al. 2012a; Field and Jones 2017; Ostafin et al. 2008). In the present study, in order to increase participants’ motivation to restrict their alcohol consumption during the assessment period, we administered a brief intervention to all participants immediately before the assessment period. During the assessment period, we measured inhibitory control twice per day for 2 weeks (with a stop signal task) and planned and actual alcohol consumption, in a group of heavy drinkers. We measured inhibitory control twice per day because multiple assessments increase reliability (Shiffman et al. 2008) and also because this permitted us to examine fluctuations in inhibitory control *within* each day.

We hypothesised that day-to-day fluctuations in inhibitory control would predict day-to-day variation in alcohol consumption after controlling for typical alcohol consumption, planned consumption on that day (see Muraven et al. 2005b), and subjective craving and mood. More precisely, we predicted that reduced inhibitory control would predict increased alcohol consumption. We also hypothesised that this relationship would be stronger when participants reported experiencing strong temptation to drink, as this would require them to actively engage inhibitory control in order to resist the urge to drink (c.f. Marhe et al. 2013). As a secondary hypothesis, we investigated if any *fluctuations* in inhibitory control within the day (i.e. the difference between the first and second assessments on each day) would predict alcohol consumption. More precisely, we predicted that more pronounced deterioration in inhibitory control over the course of the day would predict increased alcohol consumption later that day, as one might hypothesise on the basis of the resource model of self-control depletion (Baumeister et al. 2007).

## Methods

### Participants

We recruited 100 (54 female) heavy drinkers (mean 35.69 ± 9.22 years old) who expressed an interest in reducing their alcohol consumption, from the local community. Heavy drinking was defined as consumption in excess of UK government guidelines, which were < 21 units (1 unit = 8 g alcohol) for men and < 14 units for women, per week (Edwards 1996). Note that these guidelines were reduced to 14 units for both men and women, after recruitment for this study had ended (Department of Health 2016). Additional inclusion criteria were age between 25 and 65 and fluent English speaking. Participants were excluded if they had a current or previous diagnosis of substance (including alcohol) use disorder or attention deficit hyperactivity disorder, assessed using self-report. The study was advertised around the University of Liverpool campus and the wider Merseyside area via advertisements placed in newspapers and on local radio. The study was approved by The University of Liverpool research ethics committee.

### Stop signal task (see Fig. 1)

The stop signal task (Verbruggen and Logan 2008) was programmed in JAVA® for presentation on Android smartphones. The screen background was white, and all text was presented in black. On go trials, one of two go stimuli (the letter X or O) was presented, and participants were instructed to categorise the stimulus by pressing either a left or a right box that were presented on the bottom of the touch screen, as quickly as possible. This categorisation response was uninterrupted on 75% of trials. The remaining 25% of trials were ‘stop’ trials: A visual stop signal (two horizontal red lines: ‘=’) was superimposed over the image after a variable stop signal delay (see below) after onset of the go stimulus. Participants were instructed to inhibit their response on trials when the stop signal was presented.

**Fig. 1:**
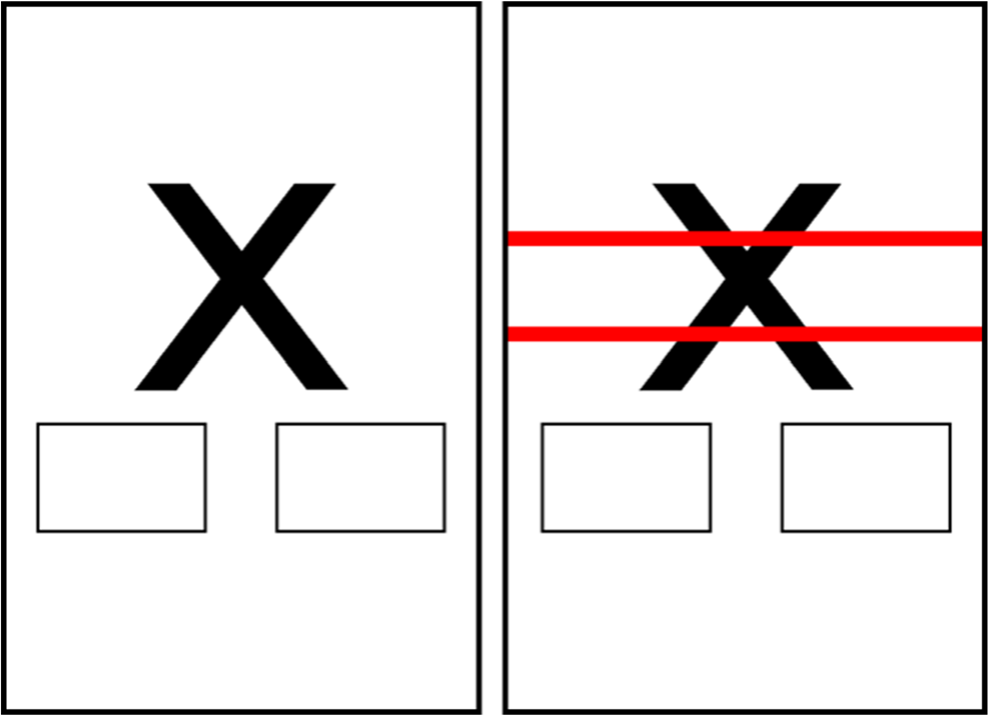
Screenshots of a go and stop trial during the stop signal task. The left panel depicts a go trial; the right panel depicts a stop trial. The two rectangles under the go stimulus were areas of the screen that participants had to press on go trials. On stop trials (right panel), the stop signal was superimposed over the go stimulus

We used a tracking algorithm to set stop signal delays (Verbruggen and Logan 2008). In each session, the first delay was set at 250 ms following the onset of the go stimulus. If participants were able to successfully inhibit responding on a given stop trial, the stop signal delay increased by 50 ms on the subsequent stop trial, which made inhibition more difficult. If participants failed to inhibit responding on a given stop trial, the delay decreased by 50 ms, which made inhibition easier (min delay = 0 ms, max delay = 1250 ms). Each assessment included three blocks of 64 trials (192 trials, 48 stop trials in total). Feedback was given on the first 16 trials of each session when participants made an incorrect response (‘*you pressed the wrong button*’ (go trials) or ‘*you should not have pressed*’ (stop trials)).

### Procedure

Participants were briefly screened by phone or email prior to attendance. Two participants were excluded from participation due to a self-reported previous diagnosis of substance use disorder. Eligible participants attended an initial appointment at a university laboratory where they provided informed consent. They then completed a questionnaire battery consisting of a timeline follow-back diary (TLFB; Sobell and Sobell 1992) to measure their alcohol consumption in the previous 2 weeks and the Alcohol Use Disorders Identification Task (AUDIT; Saunders et al. 1993) to measure hazardous drinking. The Barratt Impulsiveness Scale (BIS; Patton et al. 1995) was included to measure self-reported impulsivity, and the Temptation and Restraint Inventory (TRI; Collins and Lapp 1992) was included to measure drinking restraint. The TRI consisted of two higher order subscales: cognitive behavioural control (CBC), measuring restriction of alcohol intake, and cognitive emotional preoccupation (CEP) measuring temptation to drink.

Participants then received a brief alcohol intervention through the ‘Down Your Drink’ website (https://www.downyourdrink.org.uk/; Linke et al. 2004), the purpose of which was to increase their motivation to reduce their alcohol consumption over the course of the study period. This intervention provided participants with feedback about their current level of drinking combined with advice on cutting down, and it took approximately 30 min to complete. Participants were then loaned a smartphone (Samsung Galaxy ACE: 3.5-in screen size) preloaded with the stop signal task. The experimenter informed participants how to navigate the application and complete the task. To ensure that they understood the task, participants completed a practice block in the laboratory whilst the experimenter observed and advised, and they were able to repeat this until they felt comfortable with the task. Before leaving the laboratory, participants were given a printed guide to the number of units of alcohol in typical beverages, which they took away with them.

Participants were instructed to initiate the application between 6 a.m. and 10 a.m. every morning (morning assessment). The first time they initiated the application each day, they were prompted to answer a question about their alcohol consumption on the previous day (‘*How many units of alcohol did you consume yesterday?*’) followed by a question about their intended alcohol consumption for the current day (‘*How many units of alcohol do you plan to consume today?*’), both of which required a numerical response on a keypad displayed on the touchscreen. For random assessments (RAs), we used an automated text messaging system to send two text prompts at random intervals, once between 10 a.m. and 2 p.m. (early RA) and once between 2 p.m. and 6 p.m. (late RA), with at least 1 h between the two. Participants were required to initiate the stop signal task within 30 min of receiving the text message. During each RA, participants initially responded to a series of mood questions on visual analogue scales (VAS; ‘*How [energetic / sad / drowsy / happy] do you feel right now?*’), along with a similar question for craving (‘*How strong is your craving for alcohol right now*’), all of which had anchors labelled ‘not at all’ and ‘extremely’. Participants responded by tapping the appropriate position on the touchscreen. All VAS responses were recorded as percentages of scale length. They were also asked ‘*Have you consumed alcohol in the past 2 hours (yes, no)?*’ and ‘*Have you smoked a cigarette in the past 2 hours (yes, no)?*’ Participants then completed the stop signal task. Following completion of the task, they were asked ‘*Was there any disturbance while you were completing your entry (None / Distraction / Interruption)?*’ To ensure maximum yield of data, if participants failed to complete the morning assessment, they were also prompted to report the number of alcohol units consumed the previous day and their planned consumption for that day during the first random assessment of the day. Participants were also instructed to initiate the application at any time if they felt a strong urge to consume alcohol but had not initiated drinking (event contingent temptation assessment (TA), see Waters et al. (2012)).

After 1 week, participants returned to the university to check compliance and extract data from the smartphones. If they had completed fewer than 75% of RAs at this stage, they were withdrawn from the study; otherwise, they completed a second week of assessments before returning the phone. Upon returning after the second week, participants were asked to rate their *motivation* (‘*How motivated were you to reduce your alcohol consumption during the past 2 weeks?*’) and their *ability* (‘*How would you rate your ability to reduce your alcohol consumption during the past 2 weeks*?’) to reduce their intake during the study period on a 0 to 10 scale. Finally, participants were thanked, debriefed and reimbursed up to £150 for their participation. To increase compliance, we implemented a structured reimbursement scheme whereby the payment was contingent on the number of RAs completed, as is typical with EMA studies (see Serre et al. 2012).

### Data reduction and analysis

Go reaction times that were faster than 200 ms, slower than 2000 ms and then if more than 3 standard deviations above the individual mean were treated as outliers and removed prior to analysis (Verbruggen and De Houwer 2007). SSRT at each RA and TA was calculated using the mean method, which involves subtracting the mean SSD from mean go reaction time across the three blocks of the task (Verbruggen and Logan 2008). To determine whether participants were completing the stop signal task as required, we examined speed accuracy trade-offs (the relationship between stop signal errors and go reaction times across each RA). There were strong correlations at each RA indicative of a robust speed-accuracy trade-off. Across all participants, days and assessments (early and late), the correlation was *r* = − .904, *p* < .001. This effect was seen at every single RA: Split by day and assessment, the largest correlation was *r* = − .934, *p* < .001 (day 2, early assessment) and the smallest was *r* = − .863, *p* < .001 (day 6, early assessment). Finally, we computed the mean SSRT for each day and examined test-retest reliability, which was excellent (Cronbach’s *α* = .96; McDonalds’ *ω* = .96).

For data analysis, we used a bootstrapped (500 samples) multilevel modelling approach in MLwiN (Rasbash et al. 2009). Multilevel modelling is the most appropriate method for analysing repeated measures, nested data as it takes into account the dependence between observations due to clustering of data (for example, an individual participant’s stop signal task performance may fluctuate over time, but it should still be highly correlated with their performance at other time points). We centred the daily-level data against the mean for that individual leaving only the deviation of each measure, as recommended (Enders and Tofighi 2007). The use of multilevel models also allows for unequal number of data points across participants (due to missing data) (Hayes 2006; Quené and Van Den Bergh 2004). Our sample size was large enough (*N* > 50) to ensure robust and unbiased estimates of error (Maas and Hox 2005). We included measures of craving and mood (Serre et al. 2015), self-reported impulsivity (Dick et al. 2010) and drinking restraint (Collins et al. 2000) as additional covariates because previous research has demonstrated that these variables are associated with alcohol use. We also included age on the basis of previous reports of age-related decline in inhibitory control (Smittenaar et al. 2015), and indeed, there was a significant relationship between age and SSRT in our sample (*B* = 1.85, SE = 0.56, *p* < .01). In supplementary materials, we also report the outcomes from sensitivity analyses in which we repeated these analyses after removing data points in which go RT was an outlier in comparison to the mean go RT for that participant; findings indicated that the primary findings reported here were robust to these sensitivity analyses.

## Results

### Participant characteristics (Table 1)

Participants reduced their alcohol consumption from a mean of 66.37 (± 27.18) units in the 2 weeks prior to the study, to 55.39 (± 39.95)^1^ during the 2-week study period. This overall reduction of 10.98 units (95% CIs 2.83–19.15) was statistically significant (*t* (99) = 4.11, *p* < .001). We note that scores on the TRI CBC scale were relatively high compared to previous samples of alcohol consumers recruited from the local community (25.08 vs 19.84, see Collins et al. 2000). Taken together, this demonstrates that our participants were motivated to reduce their alcohol consumption, and they were able to do so over the course of the study period.

**Table 1:** Participant characteristics and measurements from early and late random assessments and temptation assessments. Values are means (standard deviations)

Participant-level baseline			
Age (years)	35.69 (9.22)		
AUDIT	15.08 (5.19)		
BIS total	68.11 (10.41)		
TRI CEP	34.81 (13.65)		
TRI CBC	25.08 (7.94)		
Motivation to reduce cons.	7.22 (1.63)		
Ability to reduce cons.	6.41 (1.96)		
Abstinent days	3.82 (2.67)		
Alc cons. drinking day	7.67 (4.78)		
Participant-level assessment period (2 weeks)			
Abstinent days^a^	6.28 (2.94) (range 1–12)		
Alc cons. drinking day	7.47 (5.92) (range 1–40)		
Daily level	Early RA	Late RA	TA
SSRT	318.20 (73.09)	315.86 (76.74)	290.42 (62.98)
Craving	17.71 (20.72)	28.64 (26.64)	73.10 (17.43)
Energetic	53.81 (20.75)	52.47 (20.50)	58.37 (24.77)
Sad	22.83 (20.47)	22.49 (18.50)	22.13 (21.22)
Drowsy	31.22 (23.37)	32.84 (23.13)	28.47 (24.22)
Happy	59.08 (16.87)	59.84 (16.99)	60.32 (21.67)

*Alc cons*. self-reported units of alcohol consumed in the 2 weeks prior to the study period, *Abstinent days* mean number of abstinent days during assessment period, *Alc. cons. drinking day* number of units consumed on non-abstinent days, *AUDIT* Alcohol Use Disorders Identification Test, *BIS* Barratt Impulsivity Scales, *TRI* Temptation and Restraint Inventory, *CEP* cognitive emotional preoccupation, *CBC* cognitive behavioural control, *RA* random assessment, *SSRT* stop signal reaction time, *TA* temptation assessment

^a^For days in which alcohol consumption was not recorded (5.9%), we assumed that these were drinking days.

### Compliance

Participants completed 1282 (of a possible 1400) early RAs and 1283 (of a possible 1400) late RAs within the 30-min time window. Three participants were withdrawn from the study after the first week for not completing the required 75% of assessments (although their data were retained for analysis). Participants reported drinking alcohol up to 2 h before 84 RAs (3.27%; 35 early RAs and 49 late RAs) and smoking tobacco or e-cigarettes before 56 RAs (2.18%; 15 early RAs and 41 late RAs). Participants reported being distracted or interrupted during 786 RAs (30.64%, 394 early RAs and 392 late RAs); 96 participants (96%) reported being distracted during at least 1 RA over the study period.^2^ Data from RAs that may have been confounded by alcohol intoxication, smoking, distraction or interruption (889 data points, 34.66% overall; 436 early RAs and 453 late RAs) were not included in data analyses. However, when these data points were retained in analyses, the findings reported here were unaffected, as detailed in supplementary materials. Thirty-seven participants (37%) initiated at least 1 TA, and there were 49 TAs in total; distractions/interruptions were reported during 11 (22.45%) TAs.

### Multilevel model predicting alcohol consumption during RAs (Table 2)

Our dependent variable was the total number of units of alcohol consumed on that day, as inferred from the recall questions in the morning of the subsequent day. Mean daily alcohol consumption was *β*_0_ = 3.62 units (SE = 0.26, 1 UK unit = 8 g pure alcohol). A baseline model was fitted to examine the effects of stratification of alcohol use data into levels. The one-level model consisted of the effects of assessment days, whereas the two-level model consisted of assessment days nested within individuals, with the addition of a random intercept for each individual. The two-level model was a better fit to the data than the single-level model (*χ*^2^ (1) = 123.57, *p* < .001). The majority of variance (84%) was in daily, rather than participant-level alcohol consumption according to the intra-class correlation coefficient (*r* = .840).

**Table 2:** Multilevel model examining participant-level and daily-level predictors of alcohol consumption

	Estimate (SE)	LB-CI	UB-CI
Participant level			
Alcohol cons.	.032 (.008)**	.017	.047
AUDIT	− .076 (.054)	− .181	.029
Age	.028 (.024)	− .019	.071
Motivation to reduce cons.	− .380 (.153)**	− .679	− .081
Ability to reduce cons.	− .272 (.130)**	− .526	− .018
BIS total	− .024 (.024)	− .071	.023
TRI CBC	.025 (.033)	− .039	.089
TRI CEP	.024 (.024)	− .023	.070
Daily level			
Planned	.816 (.028)**	.762	.870
Craving	.022 (.005)**	.013	.031
SSRT	.001 (.002)	− .002	.004
Energetic	.003 (.008)	− .012	.018
Sad	.011 (.008)	− .004	.026
Drowsy	.005 (.007)	− .008	.018
Happy	.028 (.010)**	.009	.047

Lower bound (LB) and upper bound (UB) confidence intervals were 95%

*Alcohol cons*. self-reported units of alcohol consumed in the 2 weeks prior to the study period, *AUDIT* Alcohol Use Disorders Identification Test, *BIS* Barratt Impulsivity Score, *TRI* Temptation and Restraint Inventory, *CBC* cognitive behavioural control, *CEP* cognitive emotional preoccupation, *SSRT* stop signal reaction time

**p < .01

In the multilevel model, we included planned alcohol consumption,^3^ craving, mood and SSRT as daily-level variables and previous alcohol consumption (in the 2 weeks prior to commencing the study), AUDIT scores, BIS total score, TRI CEP and CBC and self-reported motivation and ability to restrict consumption during the study period as participant-level variables. The model predicted 43.77% of the variance in alcohol consumption at the daily level and 70.52% at the participant level. Planned alcohol consumption, craving and happiness were significant daily-level predictors of alcohol consumption during the study period, whereas previous alcohol consumption, perceived motivation and ability to reduce alcohol consumption (but not TRI, BIS or AUDIT scores) were significant participant-level predictors. Most importantly, SSRT was not a significant predictor of alcohol consumption at the daily level.

### Temptation assessments

As expected, subjective craving was significantly higher during temptation assessments compared to random assessments (*β* = 49.82 (4.44); *p* < .001). There were no significant differences in energetic (*β* = 4.47 (3.73); *p* = .230), sad (*β* = 0.27 (3.47); *p* = .938), drowsy (*β* = − 2.88 (4.21); *p* = .491) or happy ratings (*β* = 0.14 (3.09); *p* = .964), or most importantly SSRT (*β* = − 23.22 (13.84); *p* = .095) between RAs and TAs. Due to the low number and unequal distribution of TAs amongst participants, we did not implement multilevel modelling data on TAs in a separate analysis. Incorporation of data from TAs into the final model (described above) did not explain any additional variance in alcohol consumption.

### Secondary analyses

#### Daily fluctuations in inhibitory control, craving and mood (Table 3)

We investigated whether the within-day *change* (fluctuation) in SSRT, craving and mood over the course of each day were able to account for between-day variance in alcohol consumption. Change scores were calculated by subtracting values at the early RA from values at the late RA. A positive value is indicative of an increase in SSRT (an impairment in inhibitory control) or an increase in craving/mood over the course of the day. The overall model predicted 44.84% of variance in alcohol consumption at the daily level and 70.53% at the participant level. As in the primary analysis, previous alcohol consumption and motivation were significant participant-level predictors. Planned alcohol consumption, increase in craving and increase in sadness were significant daily-level predictors. Most importantly, the change (increase) in SSRT was also a significant predictor of daily alcohol consumption, predicting 2.38% of additional variance in consumption at the daily level (but no additional variance, < 0.01%, at the participant level). These findings suggest that the magnitude of deterioration in inhibitory control over the course of the day is a significant predictor of day-to-day variation in alcohol consumption, above and beyond previous alcohol consumption, planned consumption for that day, motivation and perceived ability to reduce intake and corresponding changes in craving and mood.

**Table 3:** Multilevel model examining participant-level and daily-level within-day change as predictors of alcohol consumption

	Estimate (SE)	LB-CI	UB-CI
Participant level			
Alcohol cons.	.032 (.009)**	.015	.049
AUDIT	− .061 (.058)	− .174	.052
Age	.026 (.025)	− .023	.075
Motivation to reduce cons.	− .360 (.160)**	− .673	− .047
Ability to reduce cons	− .271 (.136)**	− .497	− .045
BIS total	− .019 (.025)	− .068	.030
TRI CBC	.025 (.034)	− .041	.091
TRI CEP	.026 (.025)	− .023	.075
Daily level			
Planned	.852 (.028)**	.798	.906
Craving change	.018 (.005)**	.009	.027
SSRT change	.007 (.002)**	.004	.011
Energetic change	− .008 (.006)	− .019	.004
Sad change	− .015 (.007)*	− .029	− .001
Drowsy change	− .004 (.006)	− .013	.005
Happy change	.022 (.007)**	.008	.036

Lower bound (LB) and upper bound (UB) confidence intervals were 95%

*Alcohol cons*. self-reported units of alcohol consumed in the 2 weeks prior to the study period, *AUDIT* Alcohol Use Disorders Identification Test, *BIS* Barratt Impulsivity Score, *TRI* Temptation and Restraint Inventory, *CBC* cognitive behavioural control, *CEP* cognitive emotional preoccupation, *SSRT* stop signal reaction time

*p < .05; **p < .01

We conducted supplementary analyses using the same models, with alcohol consumption on each day treated as a categorical variable (abstained or drank some alcohol). These analyses are reported in supplementary materials. To summarise, neither SSRT nor within-day fluctuations in SSRT were predictive of abstinence vs alcohol consumption later that day.

## Discussion

The aim of this study was to investigate if daily fluctuations in inhibitory control would predict daily variation in alcohol consumption in a sample of heavy drinkers who were motivated to reduce their alcohol consumption. Our primary hypothesis was not supported: After controlling for a number of established predictors of alcohol consumption, the between-day variation in inhibitory control did not predict the between-day variation in alcohol consumption. However, our secondary analysis demonstrated that the magnitude of change in inhibitory control *within* the day was predictive of alcohol consumption later on that day: The more that inhibitory control deteriorated over the day, the more alcohol participants consumed later that day.

Our findings did not support our primary hypothesis, because we observed no reliable association between inhibitory control on a given day and alcohol consumption later that day. However, secondary analyses demonstrated a more complex predictive relationship between inhibitory control and alcohol consumption later that day: On days in which participants’ inhibitory control worsened from the beginning of the day to the end of the day, they consumed more alcohol later that day compared to days in which their inhibitory control did not change over the course of the day. These findings are consistent with laboratory demonstrations that experimentally manipulated transient fluctuations in disinhibited mindsets prompt increased ad libitum alcohol consumption, and individual differences in inhibitory control are associated with the volume of alcohol consumed (Jones et al. 2011a; Jones et al. 2011b). They also directly extend research by Muraven et al. (2005b), who demonstrated that challenges to self-control (e.g. being required to ‘hold your tongue’ during a disagreement with a colleague at work) predicted consumption of alcohol above and beyond self-imposed daily limits, supporting the resource model of self-control (Baumeister et al. 2007). Whilst self-control is often poorly defined and inadequately measured, our findings extend those from Muraven et al. (2005a) with a well-validated and objective measure of inhibitory control. We can be confident that our findings are robust given the excellent test-retest reliability of our stop signal task, and also because our primary findings were unaffected by assessments that were contaminated by acute alcohol intoxication (Field et al. 2010), smoking (Wignall and de Wit 2011) and distractions (Verbruggen et al. 2014) which have been demonstrated to influence inhibitory control in laboratory studies.

We also identified a number of other significant predictors of day-to-day variation in alcohol consumption that, whilst not novel, serve to replicate previous findings and increase confidence in the sensitivity of our research methods. We demonstrated that daily intentions to drink, mood and subjective craving were significant predictors of alcohol consumption on that day. The predictive relationship between intentions and behaviour is well established (Sheeran 2002) and is particularly strong for alcohol use (*r+* = .54;(Cooke et al. 2016); however, this is the first time that this predictive relationship has been demonstrated on a day-to-day basis when both are measured using EMA. The observed relationship between positive and negative mood and subsequent alcohol consumption is also consistent with findings from previous EMA studies (Armeli et al. 2000). Finally, we demonstrated that momentary subjective craving predicted alcohol consumption later in the day, which is consistent with findings from previous EMA studies (Fatseas et al. 2015).

On average, participants consumed less alcohol during the 2-week study period compared to the 2-week period before starting the study, and indeed, the strength of their self-reported motivation to reduce their drinking was a robust and independent predictor of their daily alcohol intake during the study period. However, it is impossible to disentangle the cause of this reduction in drinking; it could be wholly or partially attributable to effects of the Brief Alcohol Intervention that participants received during the initial laboratory visit (Linke et al. 2004), or self-monitoring of behaviour which has been shown to be an effective behaviour change technique for reducing alcohol consumption (Michie et al. 2012). Alternatively, we may have observed a Hawthorne effect in which participants reported drinking less because they were conscious of being monitored (Jenkins et al. 2009). Additional possibilities are that measurement of alcohol consumption via daily EMA assessments may provide more accurate estimates when compared with longer term retrospective recall (Monk et al. 2015), leading to the observed differences.

Previous EMA studies with drug-dependent patients demonstrated that cognitive biases measured during participant-initiated ‘temptation episodes’ were elevated (Waters et al. 2012) and predictive of subsequent relapse (Marhe et al. 2013). Based on resource models of self-control (Baumeister et al. 2007), we would expect inhibitory control to be worse during temptation episodes compared to random assessments (Muraven et al. 2002). Despite this, we did not observe any differences in SSRT between TAs and RAs, and inclusion of TAs in our predictive model did not significantly influence the results. Therefore, our data suggest that not all cognitive variables change in line with self-reported temptations to drink. However, participants initiated far fewer TAs compared to previous EMA studies, which might be explained by differences in the populations examined. Waters et al. (2012) examined TAs in heroin-dependent inpatients, and this study took place in an inpatient detoxification centre, whereas the sample reported here were heavy drinkers recruited from the local community who were attempting to cut down. We speculate that individuals in a detoxification centre would experience more frequent and stronger temptations to use because they are less able to act on temptation compared to participants in the present study, who were able to drink alcohol at any time. Unfortunately, the small number of TAs limited our statistical power to fully examine any comparisons with RAs (Maas and Hox 2005).

The current study had a number of limitations. First, we measured the total amount of alcohol that participants consumed each day, but we were unable to infer when participants started drinking, or the rate at which they drank alcohol. Future research could ask participants to access the smartphone each time they consume an alcoholic beverage (see Collins et al. 2003). Second, asking individuals to report their ‘planned’ consumption may have led to individuals creating ‘limits’ which may in turn contribute to atypical drinking behaviour (Collins et al. 1994; Muraven et al. 2005a); similarly, this may have led to biases in self-reported alcohol use (Davis et al. 2010). Third, we allowed participants up to 30 min in which to complete a RA, which of course increases the risk that we were unable to capture *immediate* fluctuations in inhibitory control (i.e. participants may have delayed their response if they felt they would perform poorly on the task). Fourth, we only administered two assessments per day, and the inclusion of more assessments would allow us to better examine dynamic changes in inhibitory control, mood and craving. Fifth, we did not measure internal and external factors which may influence inhibitory control such as direct exposure to alcohol-related cues (see Fatseas et al. 2015); therefore, we were unable to identify the psychological triggers that may have caused inhibitory control to fluctuate throughout the day. Sixth, we were unable to cross-validate performance on our mobile stop signal task with a laboratory task. However, we note that our mobile task demonstrated excellent internal reliability and was sensitive to external variables that have been shown to influence performance on the task in laboratory settings (e.g. alcohol intoxication, see supplementary materials). Furthermore, our SSRT estimates were comparable to those reported in the broader literature (Gauggel et al. 2010; Hsieh and Lin 2017; Kok et al. 2004), and previous research has provided validation of EMA approaches to cognitive assessment (Tiplady et al. 2009). Nonetheless, future EMA studies should attempt to cross-validate the stop signal task administered on smartphones with the same tasks administered in more controlled settings. Finally, we deliberately exposed all participants to a brief alcohol intervention in order to increase their motivation to restrict their alcohol consumption for the duration of the assessment period, in accordance with theoretical arguments and previous laboratory studies (Hofmann et al. 2012; Wiers et al. 2010; Christiansen et al. 2012a; Field and Jones 2017; Ostafin et al. 2008). Nonetheless, this raises the possibility that the observed associations between inhibitory control and alcohol intake may not generalise to heavy drinkers who are *not* motivated to reduce their alcohol consumption.

To summarise, this study found evidence that within-day but not between-day fluctuations in inhibitory control were able to predict unique variance in day-to-day alcohol consumption in a sample of heavy drinkers who were motivated to reduce their consumption. Whilst the amount of variance explained was relatively small, these findings suggest that variability in objectively measured inhibitory control may be a risk factor for drinking more alcohol than planned in the near future.

## Electronic supplementary material

ESM 1 (DOCX 33 kb)

## Footnotes

1 On days in which participants did not access the device at all (5.56%), we were unable to record alcohol consumption; therefore, we imputed the individuals’ mean daily alcohol consumption as the missing value.

2 SSRT was slower (poorer inhibitory control) when participants reported distractions. There was also weak statistical evidence that SSRT was slower on assessments in which participants reported drinking any alcohol up to 2 h beforehand (see supplementary materials).

3 Repeating the multilevel model without planned alcohol consumption as a predictor did not substantially alter the results reported here.
