# Supplementary material for: Do daily fluctuations in inhibitory control predict alcohol consumption? An ecological momentary assessment study
Source: Psychopharmacology (Berl). 2018 Mar 1;235(5):1487–96. doi: 10.1007/s00213-018-4860-5 (PMC5919991; doi:10.1007/s00213-018-4860-5)
Supplement: Supplementary file 1 — (DOCX 33 kb) [file 213_2018_4860_MOESM1_ESM.docx]

**Do daily fluctuations in inhibitory control predict alcohol consumption? An ecological momentary assessment study (Supplementary materials)**

Andrew Jones^1,2^🖃, Brian Tiplady^3,4^, Katrijn Houben^5^, Chantal Nederkoorn^5^, Matt Field^1,2^

***Sensitivity analyses***

*Retention of RAs that may have been influenced by recent alcohol consumption, smoking and distractions in the analyses*

To examine the robustness of our findings we repeated the analyses after including assessments that were confounded by alcohol consumption or smoking in the preceding two hours, distractions or interruptions. We repeated the analysis of between-day variations in inhibitory control (see Supplementary table 1), and the analysis of within-day change (see Supplementary table 2). Notably, our primary findings were unaffected by these changes. The only difference was that perceived ability to reduce alcohol consumption was no longer a significant predictor of consumption in either analysis.

*RAs with outlying reaction times on Go trials.*

We repeated the analyses after removing RAs on which mean Go RT was an extreme outlier (2.75% of RAs), as such assessments may be suggestive of distraction (that was not acknowledged by the participant), which would in turn be expected to invalidate the SSRT estimate from that RA. Outlying assessments were identified on a participant-by-participant basis on the basis of box and whisker plots. Both the analysis of between-day variations in inhibitory control (see Supplementary table 3), and the analysis of within-day change (see Supplementary table 4) were unaffected by removal of these RAs.

***Examining alcohol consumption as a binary outcome.***

To examine whether inhibitory control, mood or our participant level characteristics were significant predictors of the decision to consume alcohol (or not) on any given day, we coded alcohol consumption as a binary variable (consumed alcohol = 1; did not drink = 0). We used the same multilevel structure and variables in our models (between-day variations: Supplementary table 5, within-day variations: Supplementary table 6). To summarise, in both models the only robust predictors of the decision to consume alcohol were whether individuals planned to drink, and their age.

***Validity checks***

Based on the existing evidence regarding inhibitory control, we would expect both SSRT and Go reaction time to be impaired by acute alcohol intoxication and distractions or interruptions. We would also expect SSRT to be reduced (improved inhibitory control) following nicotine administration. To test this, we ran multilevel models with SSRT and RT as the outcome variable(s) with time (Early RA, Late RT), day, participant, recent alcohol consumption, smoking, and / or distraction / interruption as predictor variables. The multilevel models were a better fit to the data than single level models (*χ^2^*(2) > 1511.52, *p* < .001).

*Recent alcohol consumption*

We tested the prediction that inhibitory control would be impaired (greater SSRT) and Go RTs would be slower on RAs on which participants reported consuming any alcohol in the preceding two hours. There was a non-significant trend in the predicted direction for SSRT (*β =* 13.89 (SE = 7.32), *p =* .058), but no evidence of slowing of Go RTs (*β =* -1.14 (SE = 13.43), *p =* .468).

*Recent tobacco smoking*

We tested the prediction that inhibitory control would be improved (faster SSRT) and Go RTs would be slower on RAs on which participants reported smoking any cigarettes or e-cigarettes in the preceding two hours. In line with our expectations, SSRT was significantly faster on RAs when participants reported smoking in the two hours beforehand (*β =* -23.76 (SE = 6.78), *p <* .001*)*. There was no evidence of slowing of RT (*β =* -21.42 (SE = 12.37), *p* = .084*),*

*Distraction*

We tested the prediction that inhibitory control would be impaired (greater SSRT) and Go RTs would be slower on RAs on which participants reported that they had been distracted or interrupted as they completed the stop signal task. SSRT was significantly slower if participants had been distracted or interrupted (*β* = 5.16 (SE = 2.43), *p =* .021), and contrary to our predictions Go reaction times (*β* = -13.88, (SE = 4.40), *p <* .001*)*, were faster on RAs on which participants were distracted or interrupted.

*Caveat on statistical power*

When interpreting these findings, it is important to acknowledge that, whilst there was significant variance at each level of these multilevel models, all were underpowered (given the small number of RAs that were preceded by alcohol consumption or were interrupted or distracted, and the small number of participants who smoked tobacco), and as a consequence all Standard Error estimates are likely to be biased (Maas & Hox, 2005). When analysing this data using single level models the only significant effects that remained is the increased SSRT on RAs on which participants reported distractions or interruptions (*β =* 7.07 (SE = 3.06), *p* = .021). Therefore, this is the only effect that is likely to be robust.

*Supplementary table 1: Multilevel model that incorporates assessments that were possibly contaminated by recent alcohol consumption or smoking, or distractions / interruptions, as predictors of between-day variation in alcohol consumption*

*Estimate (SE) LB-CI UB-CI*

*Subject level*

Alcohol cons. .033 (.008)* .017 .049

AUDIT -.085 (.050) -.181 .199

Motivation to reduce cons -.367 (.145)* -.651 -.083

Ability to reduce cons -.224 (.118) -.455 .007

BIS Total -.031 (.021) -.072 .011

TRI CBC -.001 (.030) -.060 .058

TRI CEP .047 (.021) .006 .088

Age .035 (.026) -.016 .077

*Daily level*

Planned .822 (.022)* .779 .864

Craving .033 (.004)* .024 .042

SSRT .001 (.001) -.001 .003

Energetic .004 (.006) -.008 .016

Sad .009 (.007) -.005 .023

Drowsy .000 (.000) .000 .000

Happy .032 (.007)* .018 .046

*Legend: Alcohol cons. = Self-reported units of alcohol consumed in the two weeks prior to the study period; AUDIT = Alcohol Use Disorders Identification Test; BIS = Barratt Impulsivity Score; TRI = Temptation and Restraint Inventory (CBC = Cognitive Behavioural Control; CEP = Cognitive Emotional Preoccupation); SSRT = Stop Signal Reaction Time; Lower bound (LB) and upper bound (UB) confidence intervals were 95%.*

** p < .01*

*Supplementary table 2: Multilevel model that incorporates assessments that were possibly contaminated by recent alcohol consumption or smoking, or distractions / interruptions, when predicting within-day changes as predictor variables*

*Estimate (SE) LB-CI UB-CI*

*Subject level*

Alcohol cons. .037 (.008)* .021 .053

AUDIT -.084 (.052) -.186 .018

Motivation to reduce cons. -.378 (.150)* -.672 -.084

Ability to reduce cons -.195 (.142) -.473 .083

BIS Total -.025 (.022) -.068 .018

TRI CBC .002 (.031) -.059 .063

TRI CEP .046 (.024) -.001 .095

Age .034 (.027) -.018 .087

*Daily level*

Planned .885 (.023)* .840 .930

Craving change .021 (.004)* .013 .029

SSRT change .005 (.002)* .001 .009

Energetic change -.003 (.005) -.013 .003

Sad change -.019 (.006)* -.031 -.007

Drowsy change -.003 (.004) -.011 .005

Happy change .007 (.006) -.005 .019

*Legend: Alcohol cons. = Self-reported units of alcohol consumed in the two weeks prior to the study period; AUDIT = Alcohol Use Disorders Identification Test; BIS = Barratt Impulsivity Score; TRI = Temptation and Restraint Inventory (CBC = Cognitive Behavioural Control; CEP = Cognitive Emotional Preoccupation); SSRT = Stop Signal Reaction Time; Lower bound (LB) and upper bound (UB) confidence intervals were 95%.*

** p < .01*

*Supplementary table 3: Multilevel model examining subject-level and daily-level predictors of alcohol consumption.*

*Estimate (SE) LB-CI UB-CI*

*Subject level*

Alcohol cons. .035 (.008)* .019 .051

AUDIT -.080 (.054) -.186 .026

Motivation to reduce cons. -.378 (.152)* -.676 -.080

Ability to reduce cons. -.273 (.127) -.558 .012

BIS Total -.032 (.023) -.077 .013

TRI CBC .023 (.033) -.042 .088

TRI CEP .032 (.023) -.013 .077

Age .029 (.018) -.006 .064

*Daily level*

Planned .809 (.028)* .755 .863

Craving .020 (.005)* .010 .030

SSRT .000 (.002) -.004 .004

Energetic .003 (.008) -.013 .019

Sad .010 (.009) -.008 .028

Drowsy .006 (.007) -.008 .020

Happy .023 (.010)* .003 .043

*Legend: Alcohol cons. = Self-reported units of alcohol consumed in the two weeks prior to the study period; AUDIT = Alcohol Use Disorders Identification Test; BIS = Barratt Impulsivity Score; TRI = Temptation and Restraint Inventory (CBC = Cognitive Behavioural Control; CEP = Cognitive Emotional Preoccupation); SSRT = Stop Signal Reaction Time; Lower bound (LB) and upper bound (UB) confidence intervals were 95%.*

*Supplementary table 4: Multilevel model examining subject level and daily level within-day change as predictors of alcohol consumption.*

*Estimate (SE) LB-CI UB-CI*

*Subject level*

Alcohol cons. .034 (.009)* .016 .052

AUDIT -.063 (.058) -.177 .051

Motivation to reduce cons. -.360 (.164)* -.681 -.039

Ability to reduce cons -.316 (.139)* -.588 -.044

BIS Total -.025 (.025) -.074 .024

TRI CBC .019 (.036) -.052 .090

TRI CEP .035 (.024) -.012 .082

Age .026 (.019) -.011 .063

*Daily level*

Planned .849 (.028)* .794 .904

Craving change .017 (.005)* .007 .027

SSRT change .005 (.002)* .001 .009

Energetic change -.007 (.007) -.021 .007

Sad change -.016 (.007)* -.030 -.002

Drowsy change -.001 (.011) -.023 .021

Happy change -.004 (.004) -.012 .004

*Legend: Alcohol cons. = Self-reported units of alcohol consumed in the two weeks prior to the study period; AUDIT = Alcohol Use Disorders Identification Test; BIS = Barratt Impulsivity Score; TRI = Temptation and Restraint Inventory (CBC = Cognitive Behavioural Control; CEP = Cognitive Emotional Preoccupation); SSRT = Stop Signal Reaction Time; Lower bound (LB) and upper bound (UB) confidence intervals were 95%.*

*Supplementary table 5: Multilevel model examining subject-level and daily-level predictors of daily alcohol consumption vs abstinence.*

*Estimate (SE) LB-CI UB-CI*

*Subject level*

Alcohol cons. .003 (.005) -.003 .013

AUDIT -.012 (.032) -.075 .051

Motivation to reduce cons. -.156 (.086) -.325 .013

Ability to reduce cons. -.135 (.075) -.282 .012

BIS Total .009 (.014) -.018 .036

TRI CBC -.012 (.032) -.075 .051

TRI CEP .027 (.019) -.010 .070

Age .033 (.014)* .006 .060

*Daily level*

Planned .341 (.028)* .286 .396

Craving .004 (.007) -.010 .018

SSRT .000 (.002) -.004 .004

Energetic .001 (.010) -.019 .021

Sad .010 (.011) -.012 .032

Drowsy .000 (.005) -.010 .010

Happy .021 (.012) -.003 .045

*Legend: Alcohol cons. = Self-reported units of alcohol consumed in the two weeks prior to the study period; AUDIT = Alcohol Use Disorders Identification Test; BIS = Barratt Impulsivity Score; TRI = Temptation and Restraint Inventory (CBC = Cognitive Behavioural Control; CEP = Cognitive Emotional Preoccupation); SSRT = Stop Signal Reaction Time; Lower bound (LB) and upper bound (UB) confidence intervals were 95%.*

*Supplementary table 6: Multilevel model examining subject level and daily level within-day change as predictors of daily alcohol consumption vs abstinence.*

*Estimate (SE) LB-CI UB-CI*

*Subject level*

Alcohol cons. .004 (.005) -.006 .014

AUDIT -.011 (.033) -.076 .054

Motivation to reduce cons. -.151 (.088) -.323 .021

Ability to reduce cons. -.139 (.076) -.284 -.006

BIS Total .011 (.015) -.018 .040

TRI CBC .032 (.020) -.007 .071

TRI CEP .000 (.014) -.027 .027

Age .032 (.014)* .005 .059

*Daily level*

Planned .341 (.028)* .286 .396

Craving change .005 (.007) -.009 .019

SSRT change .002 (.002) -.002 .006

Energetic change -.003 (.008) -.019 .013

Sad change .009 (.011) -.013 .031

Drowsy change .000 (.005) -.010 .010

Happy change .019 (.011) -.003 .041

*Legend: Alcohol cons. = Self-reported units of alcohol consumed in the two weeks prior to the study period; AUDIT = Alcohol Use Disorders Identification Test; BIS = Barratt Impulsivity Score; TRI = Temptation and Restraint Inventory (CBC = Cognitive Behavioural Control; CEP = Cognitive Emotional Preoccupation); SSRT = Stop Signal Reaction Time; Lower bound (LB) and upper bound (UB) confidence intervals were 95%.*
